# Higher Resting Metabolism Is Associated With Increased Free Triiodothyronine Among Female Reindeer Herders in Northern Finland

**DOI:** 10.1002/ajhb.70092

**Published:** 2025-06-20

**Authors:** Cara Ocobock, Ville Stenbäck, Alexandra M. Niclou, Päivi Soppela, Minna Turunen, Jaroslaw Walkowiak, Karl‐Heinz Herzig

**Affiliations:** ^1^ Department of Anthropology University of Notre Dame Notre Dame Indiana USA; ^2^ Eck Institute for Global Health Institute for Educational Initiatives, University of Notre Dame Notre Dame Indiana USA; ^3^ Research Unit of Biomedicine and Internal Medicine University of Oulu Oulu Finland; ^4^ US Army Research Institute of Environmental Medicine Natick Massachusetts USA; ^5^ Oak Ridge Institute for Science and Education Oak Ridge Tennessee USA; ^6^ Arctic Centre, University of Lapland Rovaniemi Finland; ^7^ Pediatric Gastroenterology and Metabolic Diseases Pediatric Institute, Poznan University of Medical Sciences Poznań Poland; ^8^ Medical Research Center Oulu University Hospital Oulu Finland

**Keywords:** cold environment, resting metabolism, thyroid hormones

## Abstract

**Background:**

Thyroid hormones (TH) regulate metabolism and are shaped by environmental factors—ambient temperature in particular. Previous work among indigenous and non‐indigenous populations in Russia revealed that there are seasonal shifts in TH dynamics such that total and free triiodothyronine (fT_3_) and free thyroxine (fT_4_) increase during winter. Elevated TH levels in these populations were positively correlated with the elevated resting metabolic rate (RMR) commonly seen among indigenous cold climate populations.

**Methods:**

Here we examined the relationship between TH levels (fT3, fT4, and thyroid stimulating hormone) and resting metabolism among reindeer herders (*N* = 35) and office workers (*N* = 16) from northern Finland in January 2019 and February of 2023. RMR was measured using indirect calorimetry at both time points and a TH analysis was conducted from venous blood samples collected before RMR measurements in 2023 only.

**Results:**

Controlling for fat free mass, female reindeer herders had significantly higher RMRs than male reindeer herders and significantly higher RMRs than predictive equation estimates. Female herders also had significantly higher fT_3_ and TSH than male herders and female officer workers. Female herders exhibited a significant positive correlation between fT_4_ and RMR; significant correlations were not found among male herders or female office workers.

**Conclusion:**

This preliminary study demonstrates variation in the relationship between TH levels and resting metabolism among reindeer herders and office workers in Northern Finland. These results highlight potential sex‐based differences in TH and metabolism dynamics, particularly among female reindeer herders, that require further research.

## 1 Introduction

Cold climate populations often display a higher resting metabolic rate (RMR, kcal/day) relative to temperate and warm climate populations as well as relative to predictive equations. This elevation can range from 3% to ~30% higher and is thought to aid in maintaining core body temperature despite cold ambient temperatures (Galloway et al. 2000; Hanna 1968; Heinbecker 1928, 1931; Katzmarzyk et al. 1994; Leonard et al. 2002, 2005, 2014; Little and Hochner 1973; Scholander et al. 1958; Snodgrass et al. 2005). Previous research has suggested that the increases in RMR seen in cold climate populations are due to higher levels of thyroid hormones (TH) (Leonard et al. 2002). In turn, THs play an important role in female reproduction and fetal development, complicating our understanding of sex differences in TH function (Krassas et al. 2010). Yet, associations between the environment, RMR, TH, and sex are not clear, limiting our understanding of potential effects on human physiology.

The activation of TH increases during cold exposure to promote heat production, in turn increasing RMR (Andersen et al. 2002; Galloway et al. 2000; Leonard et al. 2002, 2014). Cold exposure promotes the deiodination of thyroxine (T_4_) to triiodothyronine (T_3_), which stimulates the adrenergic activity of brown adipose tissue, which produces heat through non‐shivering thermogenesis (Tsibulnikov et al. 2020). Chronic or seasonal cold conditions elevate free T_3_ and free T_4_ (fT_3_, fT_4_) in humans (Andersen et al. 2012; Bojko et al. 2008). While thyroid stimulating hormone (TSH) initially stimulates thyroid follicular cells to release fT_3_ and fT_4_, it is often found unchanged in humans adapted and/or exposed to cold as its secretion is inhibited at high levels of fT_3_ and fT_4_ (Chen et al. 2016; Pirahanchi et al. 2023). However, decreased fT_3_ has been reported among cold climate populations—what is referred to as polar T_3_ syndrome, as fT_3_ is rapidly produced and taken into tissues to increase metabolism in the face of low environmental temperatures (Reed et al. 1990). This leads to an observed drop in circulating fT_3_ as the hormone becomes quickly sequestered in local tissues. For example, from summer to winter among the Indigenous Sakha of Siberia, females and males showed a decline in both fT_3_ and fT_4_ and an increase in TSH (Levy et al. 2013). Polar T_3_ syndrome has also been observed among Inuit (Andersen et al. 2012). This suggests that TH dynamics are sensitive to seasonal changes, particularly in environments for which there are drastic mean seasonal temperature differences.

There also appears to be a potential sex‐based difference in TH dynamics within a cold environment. This can be inferred from the unusual RMR pattern seen among reindeer herders in Finland, among whom females, despite weighing on average ~23 kg less, had higher absolute RMRs compared to males (Ocobock et al. 2020). Among the Evenki, an indigenous group inhabiting Siberia, a significant difference in fT_4_ between indigenous and non‐indigenous individuals was only observed among females and not males, with indigenous females displaying the higher levels (Leonard et al. 1999). The same indigenous cohort of females also displayed significantly greater RMR compared to non‐indigenous females (Galloway et al. 2000; Leonard et al. 2002). These studies examining TH dynamics in cold climates emphasize the need to evaluate fT_3_ and fT_4_ in addition to TSH, as there does not appear to be a clear and predictable pattern in response to inhabiting a cold environment. Furthermore, the observed sex differences in TH and RMR highlight the dual role of TH in the hypothalamic–pituitary‐thyroid axis (establishing and altering metabolism) and the hypothalamic–pituitary‐reproductive axis (initiating and maintaining the increased metabolic demands of pregnancy)—a dual role that may lead to unique TH patterns in cold climates (Ocobock et al. 2020).

In addition to maintaining body temperature, TH is important for tissue anabolism, especially during pregnancy (Berry et al. 1989; Silva 2009). As such, sufficient levels of TH particularly during the first 8–10 weeks of gestation, are important for maintaining a developing embryo (Fantz et al. 1999). Hypothyroidism during pregnancy is defined as having low levels of fT_4_ and elevated TSH. If TH levels do not increase ~30%–100% from baseline non‐pregnant levels so that the developing embryo experiences fT_4_ levels within the adult range, the risks of miscarriage, placental abruption, altered fetal development, fetal death, preterm delivery, low birth weight, postpartum hemorrhage, and neonatal loss are significantly elevated (Andersen et al. 2015; Klein et al. 1991; Krassas et al. 2010). Given these female physiology‐specific functions of TH, we may expect female physiology in cold climates to be more resistant than male physiology to environmental changes that could alter TH dynamics and potentially harm fertility (Ocobock et al. 2020).

We know relatively little about TH dynamics across cold populations, and how any variations in those dynamics may alter resting metabolism. Furthermore, the RMR pattern among female reindeer herders in Finland and RMR/TH dynamics among the Evenki indicate that females may have unique TH dynamics that may not be solely driven by thermoregulatory demands but also by reproductive success (Ocobock et al. 2020).

Therefore, we investigated the resting metabolism and TH levels (fT_3_, fT_4_, TSH) among reindeer herders and office workers in northern Finland. This is a preliminary study, and as such has a limited sample size with relatively few participating female reindeer herders and male office workers. Our study continues to lay the groundwork for elucidating the mechanisms underlying the association between increased RMR, TH, and sex in a cold environment. By examining TH levels, we aim to provide a potential physiological explanation for the predisposition of reindeer herder females to increased RMR.

## 2 Methods and Participant Population

### 2.1 Participants

The results of this work are part of a larger, long‐term project, Cold climate adaptations and human metabolic variation, that focuses on the cold climate adaptations and metabolic health among reindeer herders and office workers in northern Finland. This project began in February of 2023 and will continue through 2027. We also included RMR data from January of 2019 to increase the sample size of reindeer herders for analysis (Ocobock et al. 2020). The February 2023 RMR and TH analysis took place in Ivalo (68.7°N, 27.5°E) and Inari (68.9°N, 27.0°E), Finland. These locations have a mean February temperature of −11°C. The January 2019 RMR analysis, for which TH levels were not analyzed for this study, took place in Rovaniemi, Finland (66.5°N, 25.7°E, mean monthly temperature of −14°C) (Jokinen et al. 2021). From the February 2023 data, participants (*N* = 36) included reindeer herders (females *N* = 8, males *N* = 7) and office workers (females *N* = 16, males *N* = 5) from the surrounding areas. For the January 2019 data, participants (*N* = 20) included only reindeer herders (females *N* = 5, males *N* = 15). Given the extremely low number of male office workers we were able to recruit, they were removed from analysis and not reported on here. Furthermore, the number female reindeer herders with TH measures were relatively low. For analyses beyond descriptives, we compare sexes within herders and we compare between occupations for females only. For all participants, females identified as women and males as men. Sex here is defined as sex assigned at birth as identified by each participant. We will use the terms female and male. For more details about the research location and reindeer herders in northern Finland, who are both indigenous Sámi and Finns, please refer to our previous work (Ocobock et al. 2020).

Participants were recruited based on previous participation as well as through the Reindeer Herders' Association and local news outlets. The office workers in this study serve as a control to the reindeer herders, as they are typically less exposed to the cold environment and are less active due to their indoor, sedentary occupations relative to the herders. All participants were in good health and were neither pregnant nor lactating. Participants received 120€ as compensation for their participation in the study. Data collection took place at a local, unused school in Ivalo and at the dormitory for the Sámi Education Institute in Inari. All participants provided written, informed consent prior to data collection in Finnish and were provided with a detailed information sheet about the study (Heikkilä et al. 2024; Kohonen et al. 2019). The study was approved by the University of Notre Dame Internal Review Board (Protocol# 21‐09‐6826), The regional medical research ethics committee of the Wellbeing services county of North Ostrobothnia, Oulu, Finland (PPSHP EETTMK: 4/2018, POHDE EETTMK: 49/2022), and the Sámi Parliament (LAUSUNTO 25.5.2021, Dnro:34/D.a/2021).

### 2.2 Anthropometrics

We measured height and weight following standard procedure (Lohman et al. 1988). Height (recorded to the nearest 0.5 cm) and weight (recorded to the nearest 0.1 kg) were measured using a portable stadiometer (Seca Corporation, Hanover, MD) and an electronic scale (AccuWeight, New York, NY), respectively. We measured body composition using skinfold thicknesses (Lange calipers, Beta Technology, Santa Cruz, CA). We used a four‐site approach for skinfold thicknesses (bicep, tricep, subscapular, and suprailiac), which we measured on unclothed participants to the nearest to the 0.5 mm. We (CO and VS) measured each site three times and took the mean of those three measurements for analysis. We used sum of skinfolds (SoS) equations from Durnin and Womersley (Durnin and Womersley 1974) to calculate body fat percentage and fat free mass. All participants were asked to refrain from consuming alcohol 24 h before measurement, and they arrived to the data collection site 12 h fasted.

### 2.3 Thyroid Hormones Analysis

To assess TH levels, we collected whole blood venous samples, the collection of which was conducted by trained nurses from Ivalo, Finland between 08:30 and 10:00 in the morning using serum and EDTA‐plasma tubes (BD Vacutainer, New Jersey, USA). The blood samples were immediately centrifuged at 1800 g for 10 min in room temperature and the plasma portion of the sample was separated and frozen at −20°C until they were shipped on dry ice to the Research Unit of Biomedicine and Internal Medicine, University of Oulu where samples were stored at −70°C until further processing. Samples were shipped to and analyzed in the Department of Gastroenterology and Metabolism at the Poznan University of Medical Sciences in Poznan, Poland. Serum TSH, fT4, fT3, concentrations were determined by commercial kits using chemiluminescence by Abbott on an Alinity analyzer (Abbott Park, Illinois, USA).

### 2.4 Resting Metabolic Rate

We familiarized the participants with the protocol in Finnish before the measurements began. The RMR measurement was done in conjunction with an acute cooling protocol for measuring brown adipose tissue. As such, each participant wore a BCS4 whole‐body cooling suit (Med‐Eng Ottawa, ON, Canada) during this measurement. The BCS4 is a cotton sweat suit with tubing sewn into the fabric; however, no water was put into the suit during the RMR measurement. Room temperature for the measurement was a 21.8°C ± 0.7°C and 11.1% ± 2.4% humidity. Participants rested in a supine position with a small pillow under their heads and one under their knees for 30 min before any measurement took place. Once this rest period concluded, we (CO and VS) conducted RMR measurements using the mobile Cosmed K5 indirect calorimeter (Cosmed, Chicago, IL). This system employs a breath‐by‐breath measurement of oxygen consumption and carbon dioxide production to calculate RMR. We placed a mask with bi‐lateral unidirectional valves (allow inspiration through the valves, but not expiration) over the participant's mouth and nose. A mask‐based system was used rather than a hood‐based system to allow for access to the participants' supraclavicular and sternal regions for infrared measurements of surface temperature to be recorded for the brown adipose tissue measurement portion of this study not included in the present analysis.

The RMR measurement lasted 30 min, with only the last 10 min used to calculate the volume of oxygen consumed, the volume of carbon dioxide produced, and RMR (kcal/day) using the Cosmed Omnia software (Cosmed, Chicago, IL). Measured RMRs were compared to the Froehle predictive equations (Froehle 2008), referred to here as eRMR, as these were found to provide the closest approximation to actual RMR in this population (Ocobock et al. 2020).

### 2.5 Statistical Analysis

We conducted all statistical analyses using SPSS version 29 (IBM, Armonk, NY), and we produced graphical figures using Graphpad Prism (version 10.03.0). The data are normally distributed with the exception of fat free mass, for which the natural log was then used for analyses. We represent data as means ± the standard deviation and min‐max ranges. We used one‐way ANOVAs to compare age, height, weight, percent body fat, fat free mass, fT_3_, fT_4_, and TSH between females and males within reindeer herders and then between occupations for females only. We conducted multiple linear regressions to determine the effect of age, sex, and occupation on RMR, for which differences in FFM were controlled, for the January 2019 and February 2023 data combined (*N* = 55). We used paired tests to determine if measured RMR was significantly different from eRMR between sexes for herders and between occupations for females only. Pearson correlations were used to determine the relationship between RMR, fT_3_, fT_4_, and TSH for each sex and occupation for the February 2023 data only (*N* = 36).

## 3 Results

The basic anthropometric variables for herders and office workers are shown in Table 1 and the RMR and TH values in Table 2. Reindeer herders overall had significantly more fat free mass than office workers while office workers had a significantly higher body fat percentage. Reindeer herders tended to be taller and heavier than office workers; this approached but did not reach significance. Reindeer herder females were significantly shorter and had significantly less body mass and FFM than male reindeer herders, but female herders had significantly more body fat than male herders (Table 3).

**TABLE 1:** Results for age, height, body mass, body fat percentage, and fat free mass for office workers and reindeer herders.

	Age (yrs)	Height (cm)	Body mass (kg)	Body fat %	FFM (kg)
Office workers					
Female (*n* = 16)	48.7 ± 9.9	163.4 ± 5.7	69.1 ± 14.6	35.5 ± 8.0	43.9 ± 6.2
(Range)	(34–64)	(154.8–179.4)	(51.4–112.4)	(16.1–47.8)	(35.1–58.7)
Reindeer herders					
Female (*n* = 13)	34.8 ± 14.0	159.3 ± 9.0	63.8 ± 11.7	34.5 ± 4.5	41.9 ± 8.6
(Range)	(20–64)	(140.7–178.7)	(46.7–80.6)	(27.4–39.7)	(24.5–57.3)
Male (*n* = 22)	47.3 ± 10.4	179.6 ± 6.6	92.7 ± 15.5	27.9 ± 5.8	67.2 ± 11.9
(Range)	(31–64)	(169.5–194.6)	(62.9–129.6)	(15.0–35.5)	(40.7–93.7)
Mean	42.6 ± 13.2	172.1 ± 12.4	82.0 ± 19.9	30.3 ± 6.1	57.8 ± 16.3

**TABLE 2:** Summary of the resting metabolic rate, Froehle estimated resting metabolic rate, fT_3_, fT_4_, and TSH for reindeer herders and office workers.

	RMR (kcal/day)	eRMR (kcal/day)	fT_3_ pg/dL	fT_4_ ng/dL	TSH μIU/mL
Office workers					
Female (*n* = 16)	1512 ± 346	1440.5 ± 108	2.7 ± 0.23	1.0 ± 0.13	1.7 ± 0.67
(Range)	(1011–2199)	(1313–1685)	(2.24–3.09)	(0.83–1.36)	(0.57–3.20)
Reindeer herders					
Female (*n* = 13)	1694 ± 363	1450 ± 159	3.0 ± 0.26	0.89 ± 0.08	2.8 ± 1.5
(Range)	(1031–2343)	(1157–1716)	2.70–3.44 (*N* = 8)	0.76–1.00 (*N* = 8)	1.26–5.90 (*N* = 8)
Male (*n* = 22)	1870 ± 467	1882 ± 148	2.8 ± 0.32	0.87 ± 0.06	1.19 ± 0.69
(Range)	(1004–2614)	(1586–2189)	2.44–3.43 (*N* = 7)	0.79–0.95 (*N* = 7)	0.39–2.14 (*N* = 7)
Mean	1804 ± 435	1722 ± 260	2.9 ± 0.30 (*N* = 15)	0.88 ± 0.07 (*N* = 15)	2.0 ± 1.41 (*N* = 15)

**TABLE 3:** Summary of the ANOVA results comparing anthropometric and TH levels by sex between herders and between occupations for females only.

	Between occupations within females	Between sexes within herders
Height (cm)	*F* = 2.148, *p* = 0.154	***F* = 58.826**, ***p* < 0.001**
Weight (kg)	*F* = 1.207, *p* = 0.281	***F* = 33.834**, ***p* < 0.001**
FFM (kg)	*F* = 0.536, *p* = 0.470	***F* = 45.035**, ***p* < 0.001**
Body Fat %	*F* = 0.953, *p* = 0.337	***F* = 12.425**, ***p* = 0.001**
fT3 pg/dl	***F* = 7.199**, ***p* = 0.014**	*F* = 1.479, *p* = 0.246
fT4 ng/dl	*F* = 3.245, *p* = 0.085	*F* = 0.131, *p* = 0.724
TSH μIU/ml	***F* = 5.849**, ***p* = 0.024**	***F* = 6.390**, ***p* = 0.025**

*Note:* *F* and *p*‐values are provided; statistically significant results (*p* < 0.05) are presented in bold.

**FIGURE 1:**
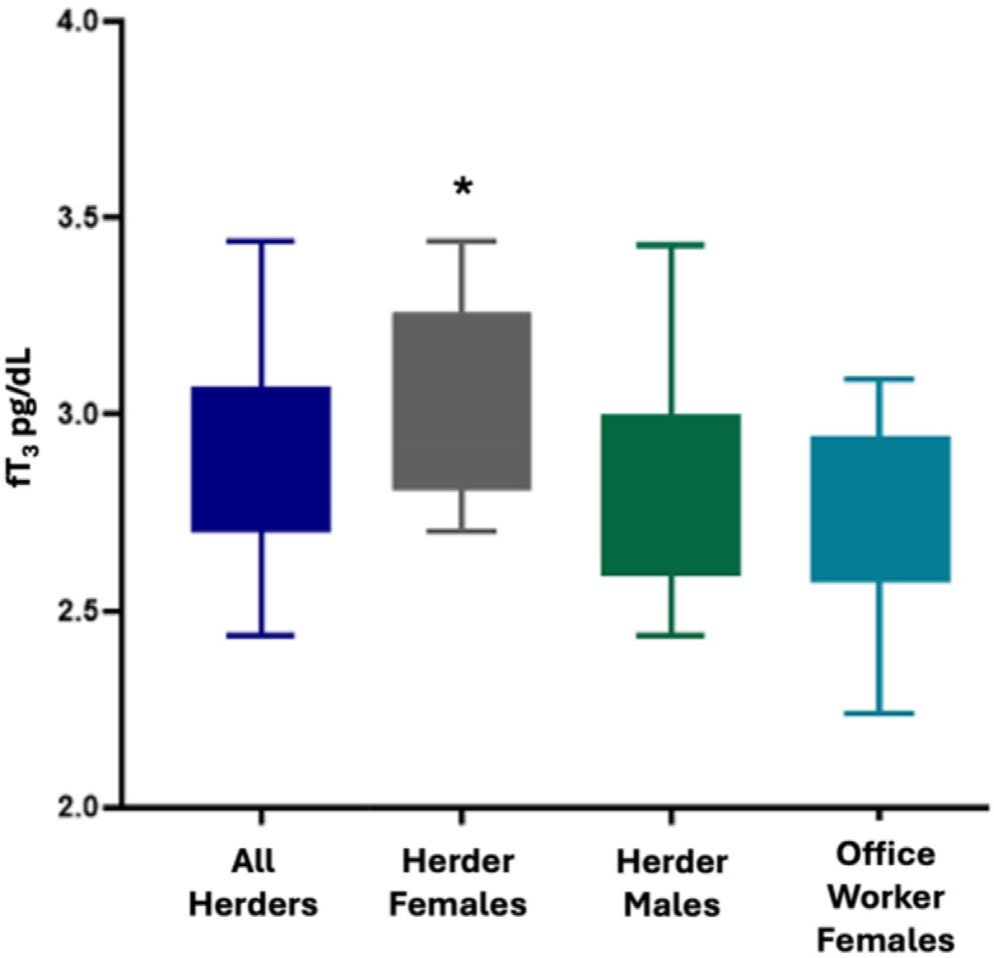
fT_3_ values by occupation and sex. Female reindeer herders had significantly higher fT_3_ than female office workers.

**FIGURE 2:**
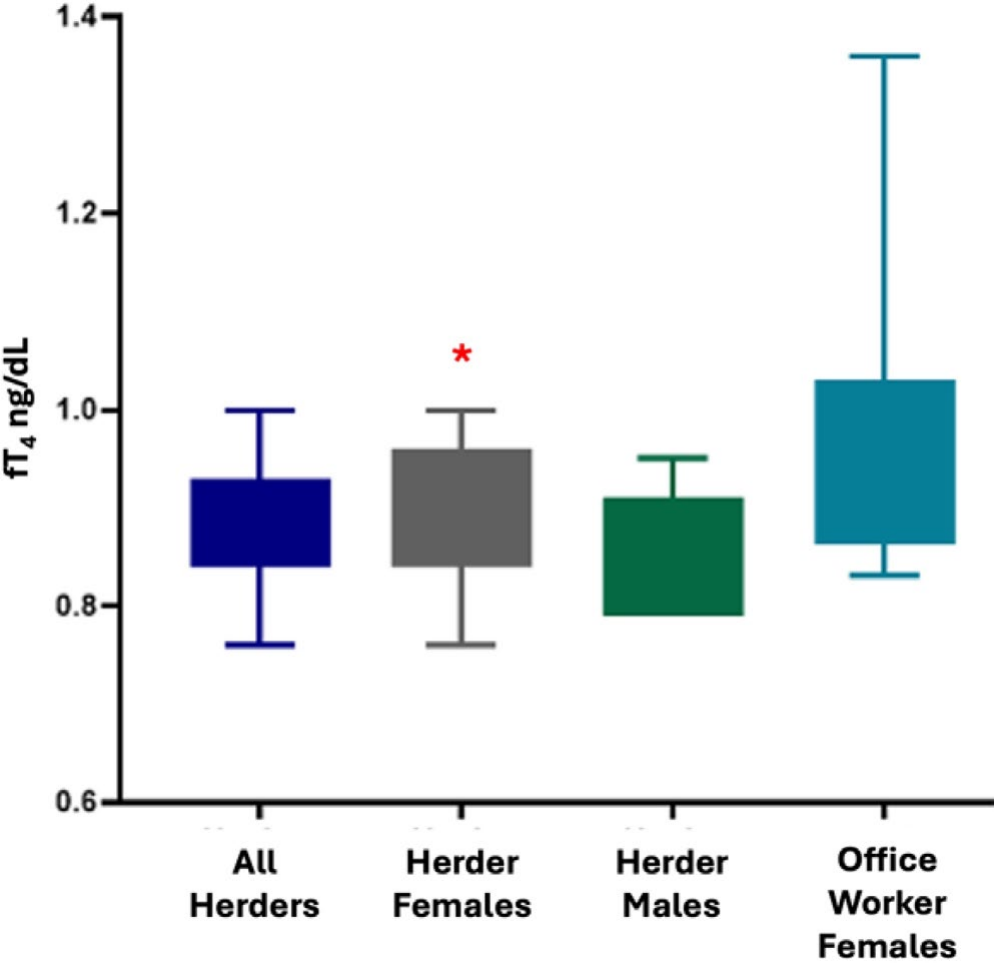
fT_4_ values by occupation and sex. Female reindeer herders displayed a positive correlation between resting metabolic rate and fT_4_.

**FIGURE 3:**
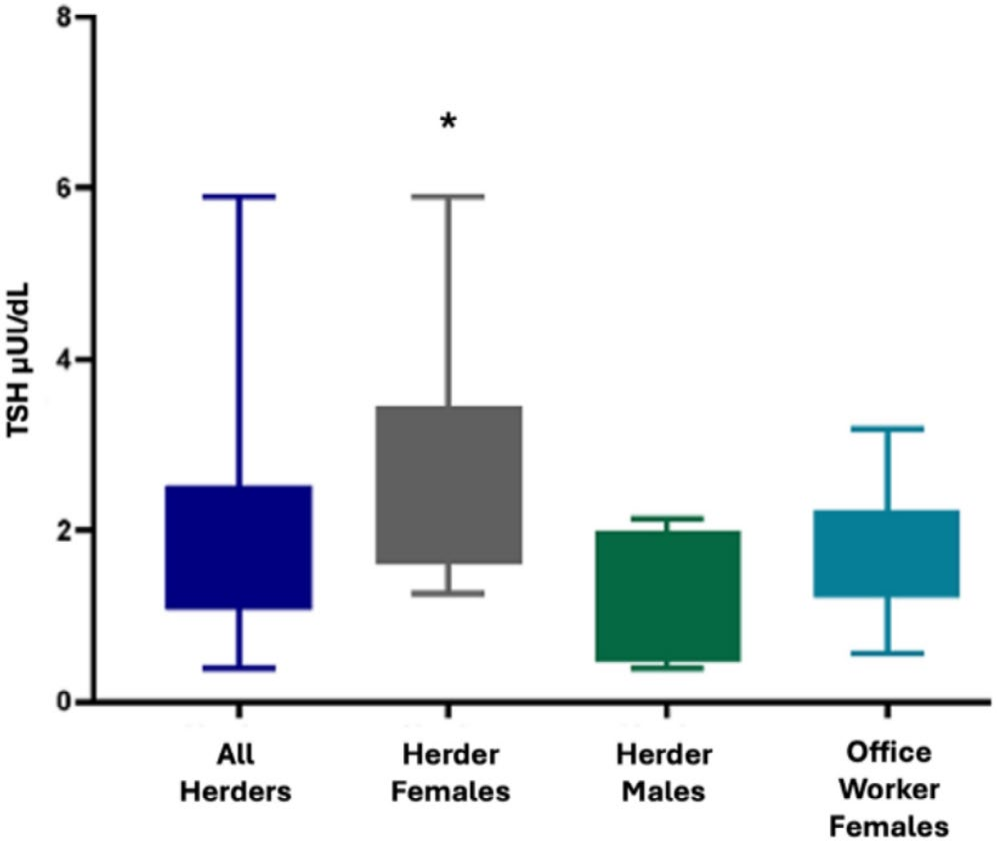
TSH values by occupation and sex. Female reindeer herders had significantly higher TSH than all other groups.

For females there were no significant differences between herders and office workers for height, weight, fat free mass, body fat percentage, or fT_4_. Female herders, however, had significantly higher fT_3_ and TSH than female office workers, and significantly higher TSH than male herders (Table 3 and Figures 1, 2, 3).

For RMR, age was not found to be a significant predictor for this dataset, so it was removed from all models. When looking within herders, there was no significant difference in RMR between the 2019 and 2023 data collection periods. Across all participants, the only significant predictor for RMR was fat free mass, though when occupation was included in the model, sex approached significance; however, being a herder or an office worker did not significantly predict RMR. When the occupations were analyzed separately, female herders once again demonstrated a significantly higher RMR, when controlling for fat free mass, than reindeer herder males. When analyzing the sexes separately, occupation was not a significant predictor (Table 4). The paired samples t‐tests revealed that only female herders demonstrated a measured RMR significantly different from and higher than eRMR (Table 5). Pearson correlations revealed that only among female herders was there a correlation between fT_4_ and RMR (Table 6).

**TABLE 4:** Multiple regression results for resting metabolic rate by sex and occupation.

	Adjusted *R* ^2^ = 0.29; *F* = 8.615; *p* < 0.001	
Resting metabolic rate regression results		
Sex	−0.251	0.068
Fat free mass	0.711	**< 0.001**
Occupation	0.094	0.156

	Adjusted *R* ^2^ = 0.338; *F* = 9.948; *p* < 0.001	
Herder RMR		
Sex	−0.540	**0.017**
FFM	0.923	**< 0.001**

	Adjusted *R* ^2^ = 0.338; *F* = 9.948; *p* < 0.07	
Female RMR		
Fat free mass	0.459	**0.030**
Occupation	0.247	0.222

*Note:* Significant results are displayed in bold. For females between occupations, included variables were fat free mass and occupation.

**TABLE 5:** Paired *t*‐test results comparing measured resting metabolic rate to estimated resting metabolic rate.

	*t*	*p*
Office females	0.913	0.376
Herder females	2.998	**0.01**
Herder males	−0.134	0.895

*Note:* Significant results are displayed in bold. Female reindeer herders had resting metabolic rates significantly higher than estimated metabolic rates.

**TABLE 6:** Pearson correlation results by sex and occupation for resting metabolic rate and thyroid hormones.

	fT3	fT4	TSH
Office worker females	−0.305, 0.251	0.107, 0.694	0.010, 0.971
Herder females	0.262, 0.532	**0.739, 0.036**	−0.257, 0.538
Herder males	0.242, 0.601	0.446, 0.316	0.247, 0.593

*Note:* Results are presented as Pearson coefficient, *p*‐value. Significant results are displayed in bold. Male office workers were excluded from this analysis due to the low sample size.

## 4 Discussion

From our previous work, we found that female reindeer herders had significantly higher RMRs than predicted and significantly higher than male reindeer herders. We hypothesized that this unexpected result was due to differences in TH dynamics between females and males driven by the differential demands of pregnancy females may incur (Ocobock et al. 2020). However, during that initial study, we were unable to assess TH levels, a deficiency we were able to presently correct. Here we analyzed the relationship between RMR and TH dynamics among a small sample reindeer herders and office workers in northern Finland.

Our present results confirm our previous findings. We found that female reindeer herders had significantly higher RMRs, when controlling for fat free mass, than their male counterparts, and male reindeer herders displayed a wide range of RMRs. However, occupation was not a significant predictor of RMR, and sex was the only significant predictor for RMR when reindeer herders were analyzed separately from office workers. Female herders had significantly higher fT_3_ and TSH than female office workers and significantly higher TSH than male herders. The only correlation found between any TH and RMR was fT_4_ among female reindeer herders.

### 4.1 TH and Cold Environments

These preliminary results add to the complex picture of the relationship between resting metabolism and TH dynamics, particularly in a cold environment. Previous work comparing the Indigenous Nenet of Russia to non‐indigenous individuals living in the same area showed an elevation in fT_4_ among both groups; however, fT_4_ was significantly more elevated in the Nenet relative to their non‐indigenous counterparts (Tkachev et al. 1991). Evenki females had significantly higher fT4 than non‐Evenki females (Leonard et al. 2002). Given the dual thermoregulatory and reproductive roles of TH, it is possible that females from cold climates may have higher baseline TH levels in addition to the compensatory TH increases associated with pregnancy or require a higher TH increase during pregnancy.

Our data partially supports this TH pattern that female reindeer herders, who typically experience greater cold exposure than their office worker counterparts, had significantly higher fT_3_, which is derived from T_4_. This is also likely a generational pattern, as reindeer herding is typically a multigenerational family endeavor. It should be noted that none of the participants in this study had fT3 or fT4 outside of the healthy range, which is 9–10 pmol/L for fT_3_ and 2.6–5.7 pmol/L for fT4 (Saltevo et al. 2016). There is no obvious explanation for the difference observed between the TH dynamics between females in these two cold climates, with the Evenki females demonstrating higher fT_4_ and the reindeer herder females from Finland demonstrating a higher fT_3_. However, there are a number of possibilities.

### 4.2 Thyroid Hormones, Socioeconomic Status, and Culture

Triiodothyronine is the more biologically active of the two TH hormones, and fT4 is actively deiodized to fT3 in the liver and kidneys. It is possible that there is a difference in kidney and liver function between these two populations. There is also a drastic difference in the standard of life in Russia in the 1990s compared to Finland in 2020, as post‐Soviet Russia experienced dramatic political, economic, and social upheaval that had detrimental effects on health and wellbeing (Abbott and Sapsford 2006). However, there is little work examining the impact of socioeconomic status and quality of life on TH levels, as a great deal more research focuses on the link between quality of life and thyroid cancer incidence. However, one study in Spain examined the relationship between income level and frequency of hyper‐ and hypo‐thyroidism. Investigators found an inverse correlation between income level and incidence of both forms of thyroid dysfunction such that those in the lowest income bracket had the highest incidence of hyper‐ and hypothyroidism (Díez and Iglesias 2023). Finally, there are cultural differences between these two groups that require further research. For example, there is a tradition in Finland of infants being placed outside to nap with only a blanket draped over the pram. This is not an observed practice in Russia, and cold exposure levels during different key developmental periods do affect adult brown adipose tissue metabolism among indigenous Siberians (Levy et al. 2021). A more detailed analysis of socioeconomic status, cultural practices throughout the life course, and TH dynamics among reindeer herders and office workers may provide greater insight into the potential confounding factor of quality of life.

### 4.3 Thyroid Hormones and Selenium Exposure

A potential confounding factor in the present study is the low selenium levels in the Finnish mountainous soil (Alfthan et al. 2015). All iodothyronine deiodinases, the enzymes that convert T4 to T3 as well other forms of TH, require selenium for proper function (Gorini et al. 2021; Mullur et al. 2014). However, Finland has taken steps to mitigate this dearth of selenium and has utilized sodium selenate fortified fertilizers since 1984 to boost selenium levels in locally grown foods and animal feed (Alfthan et al. 2015). This supplementation has brought selenium intake to optimal levels in Finland (Ruokavirasto 2023); however, this benefit is likely not felt by those who do not use these fertilizers or consume products from animals, such as reindeer, that graze on natural, unfertilized lands. Though a study in Norway did find that reindeer meat and liver are particularly high in selenium (Hassan et al. 2012). This benefit would also not be felt by those who import their own food or animal feed outside of Finland where selenium fortification does not occur. Unfortunately, there is currently no regional data on selenium intake in Finland to determine if the northern region, where our data collection occurs and which would be less likely to have selenium fortified foods, have optimal levels of selenium. Furthermore, research into the positive impact of selenium fortification has more pointedly focused on cancer occurrence and not TH levels and dynamics, as such little is known how this has impacted thyroid function. Furthermore, new European Union regulations require the use of bio‐based fertilizers rather than mineral‐based fertilizers, reducing the amount of selenium fortification, and this could have an impact on TH dynamics in the Finnish population. However, given the recency of these regulations, this has not yet been studied (Eurola et al. 2022).

### 4.4 Thyroid Hormones, Climate Change, and Sex Differences

In addition, the Arctic is experiencing climate change faster and more acutely than other parts of the world. Finland has witnessed drastic changes to seasonal weather patterns for the past 50–100 years that have not only increased mean temperatures, but also altered the timing and amount of snowfall, which used to be highly predictable. A review of these dramatic effects and the impacts they have had on reindeer herder health and occupation can be found elsewhere (Ocobock et al. 2023). Put simply, cold is no longer so cold, which can lead to noticeable changes in physiology, particularly within a cold climate population that is regularly exposed to the elements. We may well be witnessing a population in transition as it bioculturally responds to climate change. The high resting metabolism and differential TH dynamics of reindeer herding females and wide‐ranging, and, perhaps, unexpectedly low RMRs of reindeer herding males may well be evidence for this transition (Ocobock et al. 2023). What requires further exploration and explanation is the high RMRs observed among reindeer herding females.

Females, even across mammals, are less sensitive to environmental perturbations than males, often leading to more immediate detrimental effects among males than females, such as increased mortality among males (Cho et al. 2022; Kraemer 2000; Lemaître et al. 2020; Nugent et al. 2018; Seifarth et al. 2012; Zarulli et al. 2018). In the present study, the high female RMR and highly variable, relatively low metabolic rate among male reindeer herders is evidence of a potentially more steadfast physiology of females and a more sensitive physiology of males. Were females to rapidly respond to these warming environmental conditions through a decrease in TH levels and resting energy expenditure, there would likely be a negative impact on reproduction (e.g., pregnancy loss and birth defects), potentially driving the reduced sensitivity to a rapidly changing environment. This is not to say that male reproduction is not negatively impacted by greater sensitivity to the warming climate. Lower TH levels among males have been shown to alter sperm morphology; yet there does not appear to have as large of an impact on overall fertility as it does among females (Krassas et al. 2010).

### 4.5 Limitations

Though our previous results have been confirmed and resting metabolic correlations with THs established among female reindeer herders, there are several limitations to this study. First, though our sample size is far larger than our earlier work, it is still small with poor representation of male office workers, in particular. A larger sample size is needed to properly compare the sexes of the two different occupations. Second, our participant population is relatively older. Several females included were either perimenopausal or postmenopausal, which is likely to impact their TH dynamics. Third, the data presented here was collected in the winter. There are well‐documented seasonal changes in TH levels (Krassas et al. 2010; Leonard et al. 2014; Levy et al. 2013, 2016; Tsibulnikov et al. 2020). This limitation is currently being remedied as our project is now collecting seasonal data every year for three years in hopes of capturing changing metabolic and TH dynamics among reindeer herders and office workers in northern Finland. This expanded dataset will be invaluable for better understanding not only cold climate metabolism, but also the metabolic response to climate change which could have important implications for long‐term health (Gildner and Levy 2020; Ocobock et al. 2023).

## 5 Conclusion

This study examined RMRs and TH levels among reindeer herders and office workers in northern Finland. We found that female reindeer herders exhibit a pattern different from all other participants in this study such that they had higher than expected RMRs, significantly higher free T_3_ and TSH, and a positive correlation between free T_4_ and RMR. These results demonstrate that female reindeer herders are remaining physiologically resilient, potentially to protect reproductive capacity, in response to a drastically warming climate. We are also potentially witnessing a transition among male reindeer herders who are physiologically responding to the rapidly warming climate, as demonstrated by their highly variable RMRs. Finally, this work highlights the complex interaction between THs and the environment as demonstrated by the numerous environmental inputs (temperature, climate change, selenium levels, and local policy) experienced by the participants in this study. Future work will examine seasonal differences in RMR and TH dynamics as well as the impact of the menstrual cycle among herders and office workers providing greater insight into cold climate physiology and the metabolic response to climate change.

## Author Contributions

C.O., V.S., M.T., P.S., and K.‐H.H. developed this study. C.O., V.S., M.T., and P.S. carried out the data collection. K.‐H.H. and J.W. carried out blood sample analysis. C.O. carried out the data analysis. C.O., A.M.N., and V.S. wrote the manuscript. All authors reviewed the final manuscript.

## Conflicts of Interest

The authors declare no conflicts of interest.

## Data Availability

The data that support the findings of this study are available from the corresponding author upon reasonable request.
